# *CACNA1G* Causes Dominantly Inherited Myoclonus-Ataxia with Intellectual Disability: A Case Report

**DOI:** 10.1007/s12311-024-01734-6

**Published:** 2024-09-17

**Authors:** Martina De Riggi, Agnese De Giorgi, Luca Pollini, Luca Angelini, Giulia Paparella, Antonio Cannavacciuolo, Daniele Birreci, Davide Costa, Alessandra Tessa, Gemma Natale, Marco Fiorelli, Daniele Galatolo, Filippo Maria Santorelli, Serena Galosi, Matteo Bologna

**Affiliations:** 1 Department of Human Neurosciences, Sapienza University of Rome, Viale dell’Università, 30, Rome, 00185 Italy; 2 Laboratory of Neurophysiology and Plasticity, IRCCS Fondazione Santa Lucia, Rome, Italy; 3 IRCCS Neuromed, Pozzilli, IS Italy; 4 Molecular Medicine for Neurodegenerative and Neuromuscular Diseases Unit, IRCCS Stella Maris Foundation, Pisa, Italy

**Keywords:** *CACNA1G*, Myoclonus, Ataxia, Intellectual disability, Genetic

## Abstract

Spinocerebellar ataxias (SCAs) are characterized by substantial phenotypic variability. Among them, SCA42 is a rare non-expansion entity presenting with slowly progressive cerebellar syndrome but whose clinical spectrum may be also wider. A 53-year-old male presented with progressive myoclonus-ataxia and intellectual disability. Genetic screening revealed a novel c.3835G > A (p. Asp1279Asn) variant in the *CACNA1G* gene. SCA42 is a rare non-expansion SCA caused by mutations in *CACNA1G* on chromosome 17q21, encoding the Ca(V)3.1, a low-threshold voltage-gated T-type calcium channel. The novel variant we identified is potentially involved in channel activity. This case expands the knowledge regarding *CACNA1G*-associated phenotype and highlights the importance of genetic screening in myoclonus-ataxia disorders.

**Supplementary Information:**

The online version contains supplementary material available at 10.1007/s12311-024-01734-6.

## Background

Spinocerebellar ataxias (SCAs) are clinically heterogeneous hereditary neurodegenerative diseases affecting the cerebellum, the brainstem, and the spinal cord [1]. SCA type 42 (SCA42) is associated with *CACNA1G* punctuate mutations, encoding the voltage-gated calcium channel 1G, ubiquitously expressed in the brain, especially in the deep cerebellar nuclei, Purkinje cells, inferior olive nuclei and thalamus [2–4]. We here reported the case of a patient harboring a novel c.3835G > A (p.Asp1279Asn) variant presenting with progressive myoclonus-ataxia and intellectual disability.

## Case Presentation

A 53-year-old Italian man was referred to our hospital because of a progressive gait disturbance and difficulty in daily living activities.

He was born to non-consanguineous parents after an uneventful gestation and delivery. Family history was unremarkable. His sister reported that he had always had learning difficulties, but no further information could be obtained about his development. Since childhood, involuntary, irregular, and arrhythmic movements were observed in the upper limbs, that did not improve following alcohol consumption. Additionally, the patient denies experiencing any seizures.

The patient presented with an ataxic broad-based gait and an inability to walk in tandem. There was moderately dysarthric speech. The patient exhibited oculomotor disturbances: square wave jerks’ saccadic intrusion, fragmented and slow pursuit movements, and “round the house” sign in vertical saccadic movements. Involuntary, jerky, high-frequency movements were observed in the upper limbs. The patient exhibited an action tremor that worsened reaching the target (Supplementary Material 1 - Videos). On the Scale for the Assessment and Rating of Ataxia (SARA), he obtained 9/40 points. A neuropsychological evaluation demonstrated mild to moderate intellectual disability [Mini-Mental state examination (MMSE): 19.9; Montreal Cognitive Assessment (MoCA): 17/30; total IQ evaluated through Wechsler Adult Intelligence Scale – IV: 52 (Verbal Comprehension Index = 69, perceptual Organization Index = 6, working Memory Index = 66, processing Speed Index = 53)].

**Fig. 1:**
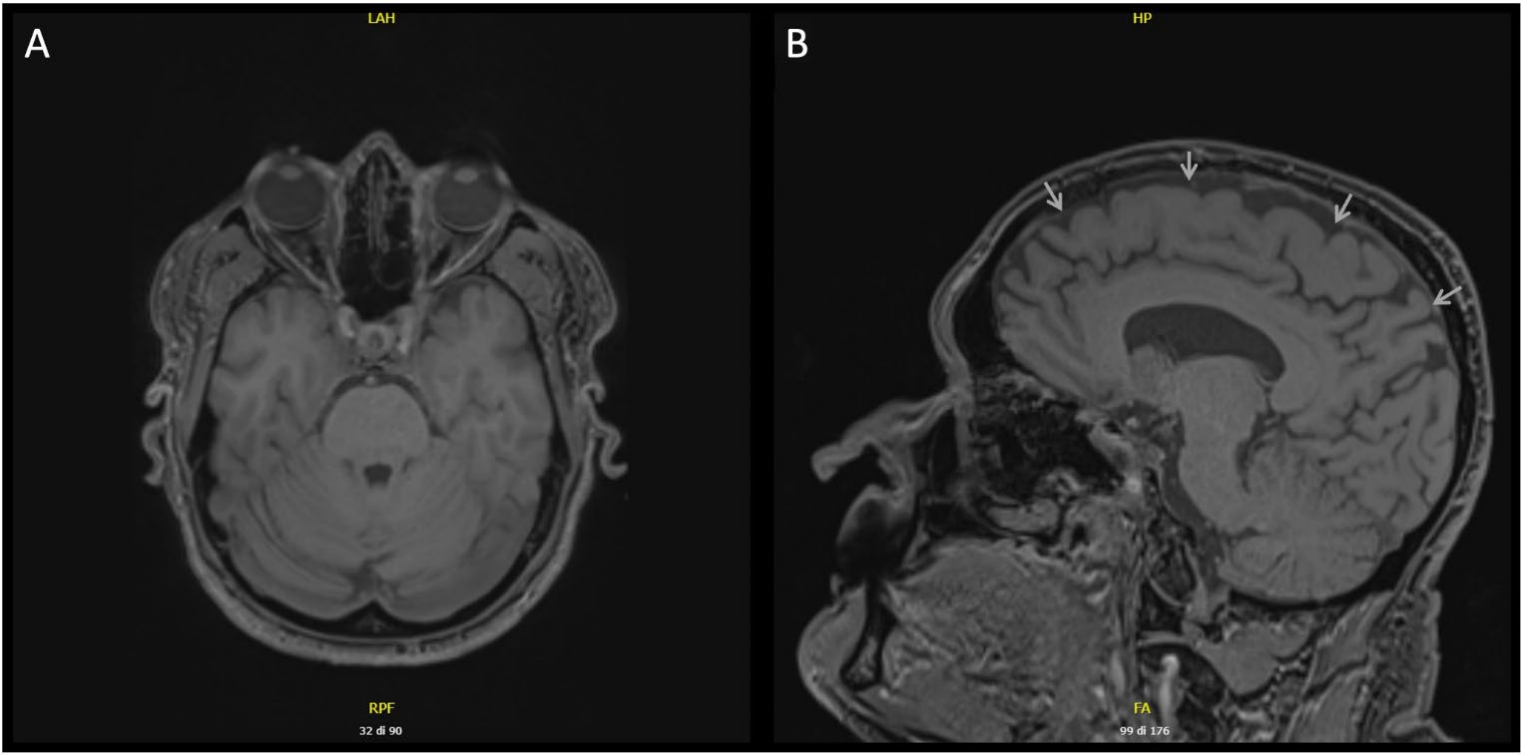
Brain magnetic resonance imaging (MRI): (**A**) T1-weighted axial image and (**B**) T1-weighted sagittal image show a slight widening of the subarachnoid spaces of the cranial vault (grey arrows) without evidence of cerebellar or brainstem atrophy

**Fig. 2:**
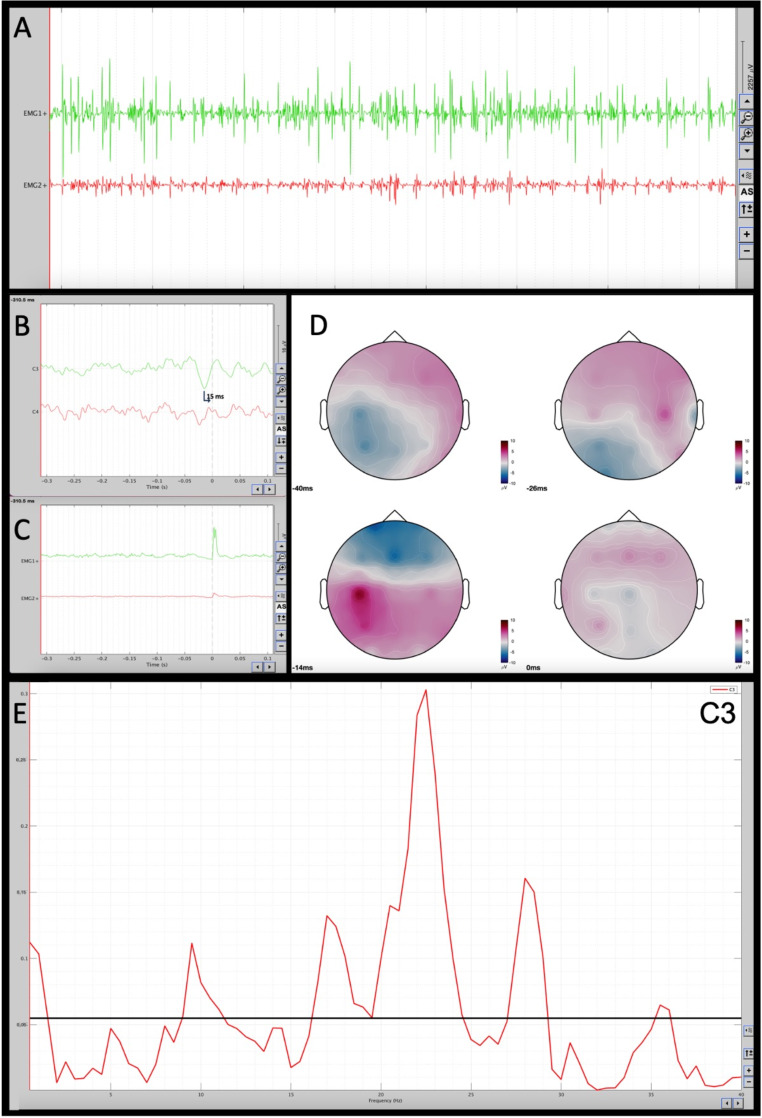
Neurophysiological assessment: (**A**) Electromyographic (EMG) trace: EMG1 + = Right wrist extensor, EMG2 + = Right wrist flexor recorded with the arms outstretched in front of the chest. The EMG trace shows frequent bursts of very short duration (30 ms) on EMG1+, which are often observed synchronously on the antagonist muscle EMG2+, compatible with myoclonus. The number of bursts per second is irregular and varies during the recording; (**B**) Jerk-Locked Back Averaging (JLBA): the JLBA performed on 71 EMG discharges at about 30 milliseconds shows a positive-negative wave on the contralateral central area (C3), with maximum positivity at -15 milliseconds from the onset of the myoclonus (time 0). No recognizable waves are seen on C4; (**C**) the averaging of the 71 myoclonic jerks, visible synchronously on EMG1 + and, with lower amplitude, also on EMG2+; (**D**) a voltage map shows the temporal trend of EEG voltages at -40, -26, -14, and 0 ms from the myoclonus. Red, white, and blue colors on the voltage map indicate respectively positive, isoelectric, and negative voltage. The highest positive voltage can be observed at -16 milliseconds over C3 area, that turns into a negative voltage at 0 milliseconds. (**E**) Cortico-muscular coherence analysis shows high peaks of coherence within the beta frequencies band (from 16 to 29 Hz) with the highest peak on 22–23 Hz. The horizontal black line indicates the 95% significant coherence level, set at 0.055. EEG–EMG data analysis was performed with Brainstorm which is documented and freely available for download online under the GNU general public license (http://neuroimage.usc.edu/brainstorm)

Brain MRI was normal, except for a slight widening of the subarachnoid spaces (Fig. 1). An electroencephalogram (EEG) showed bilateral anterior occasional slow waves with angular appearance, without clinical correlation. A surface electromyography (EMG) examination showed that the upper limbs tremolous movement was caused by frequent and arrhythmic short EMG discharges indicative of myoclonus. The cortical origin of myoclonus was revealed with jerk-locked back-averaging and cortico-muscular coherence analysis (Fig. 2). EEG-EMG analysis was performed with Brainstorm [5], which is documented and freely available for download online under the GNU general public license (http://neuroimage.usc.edu/brainstorm).

Metabolic [plasma lactate, amino acids, acylcarnitine in dried blood spot (DBS), guanidinoacetate (DBS), urinary Creatine/Creatinine ratio, urinary organic acids, urinary pterins, ceruloplasmin, plasma and 24 h urinary copper, alpha fetoprotein] and genetic studies (SNP-array, karyotype, *FMR1* pre-mutation test, and pathological expansions in polyglutamine SCA1-2-3-6-7-8-12-17) were all normal.

**Fig. 3:**
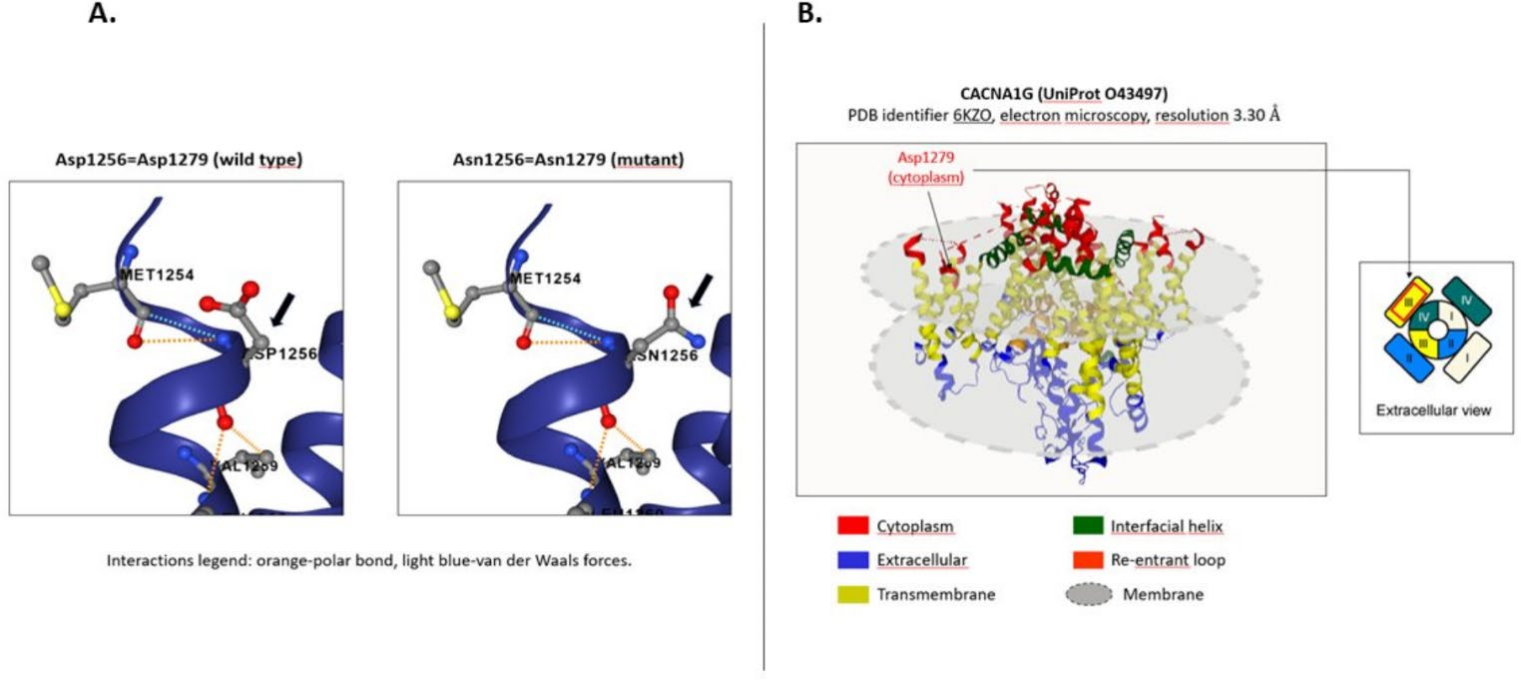
*In silico* analysis of mutated residue. (**A**) Assessment of amino acid localization was performed with Protein Data Bank of Transmembrane Proteins (PDBTM) using an experimentally determined protein structure (PDB entry: 6KZO) and revealed that aspartate in position 1279 is placed in the cytoplasmic side of S1_III_ domain. (**B**) Representation of wild-type and mutant residues within protein structure. Dotted orange line = polar bond, dotted light blue line = van der Waals forces, grey spheres = carbon atoms, red spheres = oxygen atoms, blue spheres = nitrogen atoms, yellow spheres = sulfur atoms

Massive, targeted sequencing with a panel of 285 genes (Supplementary Material 2 - Supplementary Informations) known to cause hereditary ataxias or complex syndromes in which ataxia is a symptom [6] showed a novel c.3835G > A (p. Asp1279Asn) variant in *CACNA1G*. The variant was ultrarare (gnomAD frequency 0.0001%) and predicted to be damaging *in silico* by all algorithms we tested (CADD score 31, REVEL 0.74, AlphaMissense 0.93); it was categorized as class 4/Likely pathogenic according to Varsome (https://varsome.com/, accessed on March 2024) (criteria PS4, PM2, PP3 following the ACMG guidelines [7]). *In silico* analysis of experimentally determined protein structure (8) showed that mutated residue lies in the cytoplasmic side of segment 1-repeat III (S1_III_), hence being potentially involved in voltage-sensing function (Fig. 3).

The patient was started on zonisamide 50 mg, which resulted in minimal improvement in the myoclonus and no benefit on ataxia. The addition of clonazepam (2 mg/day) resulted in a significant improvement in the myoclonus.

## Discussion

SCA42 is a rare non-expansion SCA caused by mutations in *CACNA1G* on chromosome 17q21, encoding the pore-forming alpha1 subunit of the Ca(V)3.1, a low-threshold voltage-gated T-type calcium channel organized in four repeated domains (I-IV). Each repeats is formed by four transmembrane segments (S1-S4) that compose voltage sensor domains, and two segments (S5-S6) forming the pore [9]. The most recurrent pathogenic variant (p.Arg1715His) is located in the voltage sensor S4 segment of domain IV [2, 3, 10]. This amino acid substitution results in a loss-of-function effect with subsequent decreased neuronal excitability in deep cerebellar nuclei neurons [10]. The novel c.3835G > A (p.Asp1279Asn) we identified has yet unknown functional consequences and it is located in the segment S1 of repeat III, which is part of a voltage sensor domain thus potentially impairing channel activity.

Our patient exhibited clinical features resembling those already reported in SCA42 families: a slowly progressive cerebellar syndrome characterized by ataxia, abnormal eye movements, and dysarthria [2–4, 10] (for a summary of patients with SCA42 phenotype and *CACNA1G* loss-of-function variants see supplementary Table 1). However, our patient had some distinctive features, including intellectual disability, described exclusively in two patients in the study by Coutelier et al.^8^, and high frequency action cortical myoclonus. This clinical spectrum has been observed in a subgroup of pediatric patients with syndromic infantile-onset cerebellar ataxia [11, 12]. In these patients, mutations affecting the *CACNA1G* gene have been identified, leading to a gain-of-function alteration in the Ca(v)3.1 channel [12] (Supplementary Material 2 - Supplementary Tables). In the absence of functional studies in heterologous cell system, we cannot exclude that the new variant identified fulfill both disease mechanisms, hence explaining the occurrence of manifestations seen in congenital ataxias.

Currently, there are no specific therapies available for SCA42 patients. However, some studies have hypothesized the utility of T-type calcium channel blockers in *CACNA1G* mutations patients [12]. Hara and colleagues reported benefits in a SCA42 patient from administering zonisamide, a T-type Ca channel inhibitor [13]. The myoclonus observed in our patient was only partially responsive to zonisamide and an improvement was seen with the clonazepam adjunct.

In conclusion, we expand the *CACNA1G*-associated phenotype, describing an adult patient with progressive myoclonus-ataxia and intellectual disability. Future functional studies are needed to clarify the new genetic variant neurophysiological effects.

## Electronic Supplementary Material

Below is the link to the electronic supplementary material.

Supplementary Material 1

Supplementary Material 2

## Data Availability

The data supporting this study’s findings are available on request from the corresponding author.

## Abbreviations

CACNA1G: Voltage-gated calcium channel 1G
DBS: Dried blood spot
EEG: Electroencephalogram
EMG: Electromyography
JLBA: Jerk-Locked Back Averaging
MMSE: Mini-Mental state examination
MoCA: Montreal Cognitive Assessment
MRI: Magnetic resonance imaging
PDBTP: Protein Data Bank of Transmembrane Proteins
SARA: Scale for the Assessment and Rating of Ataxia
SCAs: Spinocerebellar ataxias
SCA42: SCA type 42
SNP: Single nucleotide polymorphism
