# Supplementary material for: CACNA1G Causes Dominantly Inherited Myoclonus-Ataxia with Intellectual Disability: A Case Report
Source: Cerebellum. 2024 Sep 17;23(6):2679–83. doi: 10.1007/s12311-024-01734-6 (PMC11585495; doi:10.1007/s12311-024-01734-6)
Supplement: Supplementary file 2 — Supplementary Material 2 [file 12311_2024_1734_MOESM2_ESM.docx]

**Supplementary Informations**

Sequencing of the coding exons has been performed on the following genes:

*AARS2, ABCB7, ABCD1, ABHD12, ACO2, ADCK3, ADGRG1, AFG3L2, AHDC1, AHI1, ALDH5A1, ALG3, ALG6, AMACR, AMPD2, ANO10, APOB, APTX, ARL13B, ARSA, ATCAY, ATG5, ATL1, ATM, ATP13A2, ATP1A2, ATP1A3, ATP2B3,ATP7B, ATP8A2, BEAN1, BRAT1, BRF1, C10ORF2, C12ORF65, C19ORF12, C5ORF42, C9ORF72, CA8, CACNA1A, CACNA1G, CACNB4, CAMTA1, CASK, CC2D2A,CCDC88C, CD40LG, CDK5, CEP104, CEP290, CEP41, CHMP1A, CLCN2, CLN5, CLN6, CLN8, CLP1, COA7, COQ2, COQ4, COQ9, COX20, CP, CSPP1, CSTB,CTBP1, CTSD, CWF19L1, CYP27A1, CYP7B1, DAB1, DARS, DARS2, DDHD2, DKC1, DNAJC19, DNAJC3, DNMT1, EEF2, EIF2B1, EIF2B2, EIF2B3, EIF2B4, EIF2B5,ELOVL4, ELOVL5, ERCC4, ERCC8, EXOSC3, EXOSC8, FA2H, FARS2, FASTKD2, FAT2, FGF14, FLVCR1, FMR1, FOLR1, FXN, GALC, GAN, GBA2, GBE1, GFAP,GJB1, GJC2, GLB1, GOSR2, GRID2, GRM1, HARS, HARS2, HEXA, HEXB, HIBCH, HSD17B4, INPP5E, ITPR1, KCNA1, KCNA2, KCNC1, KCNC3, KCND3, KCNJ10,KCNMA1, KCTD7, KIF1A, KIF1C, KIF7, LAMA1, LMNB2, LYST, MARS2, MED17, MFN2, MFSD8, MKS1, MMACHC, MME, MRE11A, MTPAP, MTTP, MVK, NAGLU,NDUFS1, NDUFS7, NEU1, NOL3, NOP56, NPC1, NPC2, NPHP1, OFD1, OPA1, OPA3, OPHN1, PAX6, PCLO, PCNA, PDE6D, PDHA1, PDSS1, PDSS2, PDYN, PEX10,PEX16, PEX2, PEX6, PEX7, PHYH, PIK3R5, PLA2G6, PLD3, PLP1, PMM2, PMPCA, PNKP, PNPLA6, POLG, POLR3A, POLR3B, PPT1, PRICKLE1, PRKCG, PRNP, PRPS1, PRRT2, PSAP, PSEN1, PTF1A, PTRH2, PUM1, QARS, RAB3GAP1, RARS, RARS2, RELN, RNF170, RNF216, RPGRIP1L, RUBCN, SACS, SAMD9L, SARS,SCN1A, SCN2A, SCN8A, SCYL1, SEPSECS, SETX, SIL1, SLC17A5, SLC1A3, SLC25A46, SLC2A1, SLC33A1, SLC35A2, SLC52A2, SLC6A19, SLC9A1, SLC9A6, SMPD1,SNAP25, SNX14, SPAST, SPG11, SPG7, SPTAN1, SPTBN2, SRD5A3, STS, STUB1, STXBP1, SURF1, SYNE1, SYNE2, SYT14, TBC1D23, TCTN1, TCTN2, TCTN3, TDP1,TDP2, TGM6, TINF2, TMEM138, TMEM216, TMEM231, TMEM237, TMEM240, TMEM67, TOE1, TOP1, TPP1, TRNT1, TRPC3, TSEN15, TSEN2, TSEN34, TSEN54,TTBK2, TTC19, TTC21B, TTPA, TUBB3, TUBB4A, UBA5, UBR4, UCHL1, VAMP1, VARS2, VLDLR, VPS53, VRK1, VWA3B, WDR73, WDR81, WFS1, WWOX, XPA,XRCC1, XRCC4, ZFYVE26, ZFYVE27, ZNF423*

**Supplementary Table 1: Literature review of patients with loss-of-function CACNA1G mutations (1/2)**

|  | Morino 2015 | | Coutelier 2015 | Kimura 2017 | | Li 2018 | Ngo 2018 | |
| --- | --- | --- | --- | --- | --- | --- | --- | --- |
|  | Family 1 | Family 2 | Family AAD-SAL-233, AAD-GRE-319, AAD-SAL-454 | Family 1 | Family 2 | Family 1 | Family A | Family B |
| Examined subjects | 10 | 5 | 10 | 5 | 3 | 3 | 3 | 2 |
| CACNA1G variant | c.5144G > A;  p.Arg1715His | c.5144G > A; p.Arg1715His | c.5144G > A;  p.Arg1715His | c.5144G > A;  p.Arg1715His | c.5144G > A; p.Arg1715His | c.4721T> A;  p. Met1574Lys | c.5144G > A; p.Arg1715His | c.5144G>A;  p.Arg1715His |
| Age onset (years) | 20-70 | 18-57 | 9-78 | 13-38 | 35-36 | 43-60 | 22-58 | 39-67 |
| Age at last follow-up (years) | NOT ASSESSED | NOT ASSESSED | 28-82 | 16-40 | 57-87 | 45-70 | NOT ASSESSED | NOT ASSESSED |
| Symptoms onset | NOT ASSESSED | NOT ASSESSED | Gait instability (8 *patient)*; vertigo (1 *patient)*; gait instability and vertigo (*1 patient)* | Gait instability (4 *patients)*; poor handwriting (1 *patient*); head tremor (1 *patient)* | Gait instability | Gait instability | Subjective sense of leg weakness and imbalance | Imbalance and speech involvement |
| Ataxic gait | + | + | + | + | + | + | + | + |
| Appendicular ataxia | NOT ASSESSED | NOT ASSESSED | + 7 *patients* | + | + | + | + | + |
| Dysarthria | + | + | + *8 patients* | + *4 patients* | + | + | + | + |
| Ocular signs | NOT ASSESSED | NOT ASSESSED | Saccadic pursuit *(4 patients);*  hypometric saccades (*2 patients);* diplopia *(2 patients);* strabismus *(1 patient);* gaze nystagmus *(1 patient)* | Saccadic pursuit *(4 patients);* Ocular overshoot (*3 patients*) | Saccadic pursuit *(2 patients);* ocular overshoot *(all patients)* | NOT ASSESSED | - | NOT ASSESSED |
| Pyramidal signs | NOT ASSESSED | NOT ASSESSED | Spasticity *(3 patients),* Babinski sign *(2 patients),* Upper and lower limb iperreflexia (*4 patients)* | Spasticity (*1 patient)*, lower limb iperreflexia (*3 patients)* | - | hyperreflexia (*1 patient)* | - | NOT ASSESSED |
| Brain MRI | NOT ASSESSED | Cerebellar atrophy (patient 2-III-1) | Vermian cerebellar atrophy, cerebellar and brainstem  hypoplasia and atrophy, less  foliation of the hemispheres,  N-acetylaspartate decrease  (*5 patients)* | Cerebellar atrophy | Cerebellar atrophy | Cerebellar atrophy  (*all patients)*  “hot cross bun sign” in brainstem (*2 patient)* | Cerebellar atrophy | Cerebellar atrophy |
| Cognitive impairment/ Psychiatric symptoms | - | - | Alzheimer disease *(1 patient);* MMSE 25/30 *(1 patient);* depression *(3 patients)* | - | - | - | - | NOT ASSESSED |
| Involuntary movements | - | Resting tremor | Myokymia orbicularis *(3 patient)*  postural upper limbs and head tremor  *(1 patient)* | Head tremor (*1 patient)* | - | - | - | NOT ASSESSED |
| Other findings | NOT ASSESSED | In patient 2-III-1 low-dose zonisamide (25 mg a day) improved resting tremor and walking stability | Dysphagia or swallowing difficulties (3 patients); scoliosis (1 patient); psoriasis (1 patient) | Duodenal lymphoma *(1 patient)*; hypertension *(1 patient)* | Hearing impairing and hypertension *(1 patient)* | - | NOT ASSESSED | NOT ASSESSED |

IQ: intelligence quotient, MMSE: Mini-Mental State Examination; MRI: magnetic resonance imaging.

**Supplementary Table 1: Literature review of patients with loss-of-function CACNA1G mutations (2/2)**

|  | Ngo 2018 | Hashiguchi 2019 | Gazulla 2021 | | Riquet 2023 | Our patient |
| --- | --- | --- | --- | --- | --- | --- |
|  |  | Family 1 |  |  |  |  |
| Examinated members | 1 | 10 | Patient 1 | Patient 2 (mother of patient 1) | 1 | 1 |
| CACNA1G variant | c.5144G > A; p.Arg1715His | c.5144G > A; p.Arg1715His | c.6958G>T (p.Gly2320Cys) | c.6958G>T (p.Gly2320Cys) | c. 5152C>G, p.(Arg1718Gly) | c.3835G>A (p.Asp1279Asn) |
| Age onset (years) | 25 | 13-32 | 28 | 45 | 2 months | 10 |
| Age at last follow-up (years) | NOT ASSESSED | NOT ASSESSED | 45 | 72 | 1.5 | 53 |
| Symptoms onset | Imbalance and intermittent jerking movements | Cerebellar syndrome | Bouts of dizziness and gait unsteadiness that lasted 3 min-2 h, occurred weekly, and left residual instability during 24 h | Bouts of dizziness, gait unsteadiness, visual blurring, mental slowing and fatigue during 30 min-24 h | Involuntary, brief, and repeated upward eye deviation, without unconsciousness | Learning disability, gait disturbance, myoclonus |
| Ataxic gait | + | + | + Episodic | + Episodic | + | + |
| Appendicular ataxia | + | + | + Episodic | + Episodic | + | + |
| Dysarthria | - | NOT ASSESSED | + Episodic | NOT ASSESSED |  | + |
| Ocular signs | - | NOT ASSESSED | Impairment of the visually enhanced vestibulo-ocular reflex | Impairment of the visually enhanced vestibulo-ocular reflex | - | Square wave jerks’ saccadic intrusion, fragmented and slow pursuit movements, and “round the house” sign in vertical saccadic movements |
| Pyramidal signs | - | + Pyramidal signs (not all patients) | Knee and ankle clonus, brisk reflexes | Brisk reflexes | NOT ASSESSED | - |
| Brain MRI | Cerebellar atrophy, primarily of the vermis | Moderate cerebellar atrophy (*all patient*); cerebral and brainstem atrophy *(1 patient with dementia)* | Normal | NOT ASSESSED | Normal | Normal |
| Cognitive impairment/  Psychiatric symptoms | NOT ASSESSED | Learning disability and progressive dementia *(1 patient),* aggressive behavior and delusions *(1 patient)* | Episodic cognitive slowing | Episodic mental slowing and fatigue | Developmental delay: able to sit but not stand, no verbal language | Intellectual disability  (IQ 52) |
| Involuntary movements | Jerking movements of the neck that slowly progressed to a tremor with cervical dystonia | Truncal myoclonus *(3 patients)* | NOT ASSESSED | NOT ASSESSED | Episodes of paroxysmal torticollis (4 months).  Prolonged episodes of upward gazes during acute febrile states (8 months) | Cortical myoclonus involving upper limbs |
| Other findings | NOT ASSESSED | Patient II-3 postmortem examination: cerebellar degeneration with prominent Purkinje and granule cells loss; thinner molecular layer; myelin pallor, fibrillary gliosis, spongiform changes in cerebellar white matter | Episodes started with oppressive right frontoparietal headache, right cheek numbness, unstable gait, cognitive slowing, dysarthria, and feeling of ear fullness, improved with carbamazepine treatment | NOT ASSESSED | Topiramate allowed a significant reduction of abnormal eye movements | Myoclonus was only partially responsive to zonisamide and an improvement was seen with the clonazepam adjunct. |

IQ: intelligence quotient, MMSE: Mini-Mental State Examination; MRI: magnetic resonance imaging.

**Supplementary Table 2: Literature review of patients with gain-of-function CACNA1G mutations (1/2)**

|  | Chemin 2018 | | | | Barresi 2019 | | |
| --- | --- | --- | --- | --- | --- | --- | --- |
|  | Subject I | Subject II | Subject III | Subject IV | Subject I | Subject II | Subject III |
| CACNA1G Variant | c.2881G>A; p.Ala961Thr | c.4591A>G p.Met1531Val | c.2881G4A; p.Ala961Thr | c.2881G>A; p.Ala961Thr | c.2881G>A; p.Ala961Thr | c.2881G>A; p.Ala961Thr | c.2881G>A; p.Ala961Thr |
| Inheritance | *de novo* | *de novo* | *de novo* | *de novo* | *de novo* | *de novo* | *de novo* |
| Age onset | 3 months | 8 days | 7 days | 4 months | neonatal age | 9 months | before 1 year of age |
| Age at last follow-up (years) | 11.5 | 13 | 3 | 8 | 11 | 2.5 | 3 |
| Gender | Female | Female | Female | Female | Male | Female | Male |
| Microcephaly | + | + | - | - | + | + | + |
| Weight/  Length at birth | 2.4 kg/43 cm (-2 SD both) | 2.8 kg/49 cm (-1 SD weight) | 2.9 kg/49.5 cm (-1 SD weight) | -2.9 kg/49.5 cm (-1 SD weight) | 2 kg | 4 kg/49 cm | 3 kg |
| Psychomotor development | Delayed (can stand hand supported/  understands  simple orders) | Delayed (cannot hold her head) | Delayed (sat in tripod at 2 years 7 month / non-verbal) | Delayed (Unable to walk/ non-verbal/autistic behaviour) | Delayed (unable to walk/ non-verbal/behavioural  disorder) | Delayed (unable to sit/ non-verbal/ poor interaction) | Delayed (unable to walk/ non-verbal) |
| Cognitive impairment | Severe intellectual disability | Severe intellectual disability | Severe intellectual disability | Severe intellectual disability | Severe intellectual disability | Severe intellectual disability | Severe intellectual disability |
| Seizures | - | Epileptic  Encephalopa  -thy (8 days onset) | Epileptic encephalopa-thy (7 days onset) | - | - | - | - |
| EEG | Normal | Normal | Multifocal epileptic discharges | Slow background activity | Irregular sharp waves on the center- posterior derivations of both hemispheres, sometimes left-handed | Normal | Normal |
| Muscle tone | Axial hypotonia | Axial hypotonia and distal hypertonia | Axial hypotonia and distal hypertonia | Axial hypotonia  and distal hypertonia | Axial hypotonia and distal hypertonia | Axial hypotonia and distal hypertonia | Axial hypotonia |
| Dysmetria | + | NOT ASSESSED | NOT ASSESSED | + | NOT ASSESSED | - | + |
| Cerebellar ataxia | + | + | + | + | + | - | off balance |
| Ocular signs | Oculomotor apraxia, strabismus | Oculomotor apraxia, strabismus | Hyperopia | Oculomotor apraxia, strabismus | Esotropia, Doll’s eyes, slightly pale papillae, horizontal nystagmus | Exotropia | Strabismus |
| Brain MRI | Global cerebellum atrophy | Vermis atrophy | Global cerebellum atrophy | Global cerebellum atrophy | Progressive vermis atrophy; dentate nucleus hyperintensity | Inferior vermis hypoplasia | Mild cerebellar parenchymal volume loss |
| Dysmorphic face features | Small palpebral fissures, anteverted ears | Prognathism, synophrys | Mild hyperteloris  -m | Missing upper incisors | Frontal bossing, mild hypertelorism, eyesbrows deep-set, short palpebral fissures, large lobe ears, thin nasal pyramid, hypoplasia alae nasi, wide mouth, downturned corners of mouth, spaced teeth | Frontal bossing, mild hypertelorism, eyesbrows deep-set, anterior creases and large lobe, thin nasal pyramid, hypoplasia alae nasi, wide mouth, downturned corners of mouth, prognathism | Frontal bossing, eyesbrows deep-set, short palpebral fissures, large lobe ears, thin nasal pyramid, prominent columella, hypoplasia alae nasi, wide mouth, downturned corners of mouth, micrognathia |
| Dysmorphic hands and feet features | Short hands and feet, thin digits, clinodactyly | Short hand and feet, clinodactyly | Syndactyly of hands and toes | Broad thumbs and great toes, persistent fetal pads on fingers | Hypoplasia phalanx V finger, V finger clinodactyly, broad I thumb | V finger clinodactyly, persistent fetal fingertip pads, broad I toe and thumb | Broad I toe and thumb |
| Dysmorphic hair features | Hypotrichosis and sparse hair | Hirsutism, low posterior hairline | NOT ASSESSED | Hypertrichosis and thick hair | Thin and spared hair, high frontal hairline | Hypotrichosis, thin hair, high frontal hairline | High frontal hairline |

EEG: electroencephalography; MRI: magnetic resonance imaging; SD: standard deviation.

**Supplementary Table 2: Literature review of patients with gain-of-function CACNA1G mutations (2/2)**

|  | Barresi 2019 | Berecki 2020 | | Casas-Alba 2020 | Kunii 2020 | |
| --- | --- | --- | --- | --- | --- | --- |
|  | Subject IV | Subject I | Subject II | Subject I | Subject I | Subject III |
| CACNA1G Variant | c.4591A>G; p.Met1531Val | c.2727G > C; p.Leu909Phe | c.623T> C; p.Leu208Pro | c.2881G>A; p.Ala961Thr | c.2881G > A; p.Ala961Thr | c.4591A > G; p.Met1531Val |
| Inheritance | *de novo* | *de novo* | *de novo* | *de novo* | *de novo* | *de novo* |
| Age onset | 2 days | 7 months | 2 months | 4 months | 6 months | 3 months |
| Age at last follow-up (years) | 1 | 9 | 17 | 2 | 11 | 8 |
| Gender | Male | Female | Male | Female | Female | Female |
| Microcephaly | + | NOT ASSESSED | NOT ASSESSED | + | NOT ASSESSED | NOT ASSESSED |
| Weight/Length at birth | 3 kg/50 cm | NOT ASSESSED | NOT ASSESSED | 3.2 kg/50 cm | NOT ASSESSED | NOT ASSESSED |
| Psychomotor development | Delayed (absent language/poor interaction) | Developmental regression (walking with a walker at age 4 years and eye gaze-based communication) | Delayed (unable to sit unsupported/ absent language) | Delayed (head control at 4 months/stuttering/ unable to sit unsupported/social smile) | Developmental regression (head control at 6 months/rolling over at 18 months/stereotypic hand movements) | Delayed (cannot hold her head/ bedridden) |
| Cognitive impairment | Severe intellectual disability | Severe intellectual disability | Severe intellectual disability | Severe intellectual disability | Intellectual disability | Intellectual disability |
| Seizures | 2 days onset | Epileptic encephalopathy (7 months onset) with fever seizures, tonic-clonic seizures, and absences | Myoclonic jerks (4 months onset) | - | - | Myoclonus (3 months onset), West syndrome (8 months onset), massive myoclonic seizure (3 years onset) |
| EEG | Multifocal spikes and waves discharges | Generalized spike-wave discharges, polyspike waves, multifocal discharges, generalized paroxysmal fast activity | Multifocal spikes and sharp waves and generalized bursts of 4-4.5 Hz activity and sharply contoured 6-7 Hz activity | Normal | NOT ASSESSED | Hypsarrhythmia |
| Muscle tone | Axial hypotonia and peripheral hypertonia | Hypotonia | Axial hypotonia, appendicular spasticity, neuromuscular scoliosis | Axial hypotonia | Spasticity | Rigidity/spasticity |
| Dysmetria | Impossible to test | NOT ASSESSED | NOT ASSESSED | NOT ASSESSED | NOT ASSESSED | NOT ASSESSED |
| Cerebellar ataxia | + | + | Wheelchair-dependent | + | + | + |
| Ocular signs | Absent eye tracking, erratic eye movements | NOT ASSESSED | NOT ASSESSED | Strabismus, exotropia | NOT ASSESSED | NOT ASSESSED |
| Brain MRI | High signal intensity lesion in the mesial aspect of the left cerebellum hemisphere, mild atrophy of folia in the cerebellar cortex | Normal (8 years) | Normal (multiple times) | Widening of the interfolia spaces predominantly in the superior vermis | Cerebral atrophy, Normal cerebellum | Progressive cerebellar atrophy  Cerebral atrophy |
| Dysmorphic face features | Frontal bossing, plagiocephaly, eyesbrows deep-set, anteverted ears, prominent columella, hypoplasia alae nasi, downturned corners of mouth | - | - | Broad forehead, deep-set eyes, retrognathia, wide mouth | NOT ASSESSED | NOT ASSESSED |
| Dysmorphic hands and feet features | Syndactyly I and II finger, clinodactyly of II and V finger with bilateral hypoplasia of the middle phalanges; diffuse koilonychia | - | - | Broad hallux, diffuse clubbing of toes, ulnar deviation of the second finger, prominent interdigital folds, fifth-finger clinodactyly, and thigh lipodystrophy | NOT ASSESSED | NOT ASSESSED |
| Dysmorphic hair features | High frontal hairline, forehead hirsutism | - | - | Sparse and thin hair, high frontal hairline | NOT ASSESSED | NOT ASSESSED |

EEG: electroencephalography; MRI: magnetic resonance imaging; SD: standard deviation.

**SUPPLEMENTARY MATERIAL REFERENCES**

Barresi, S., Dentici, M. L., Manzoni, F., Bellacchio, E., Agolini, E., Pizzi, S., Ciolfi, A., Tarnopolsky, M., Brady, L., Garone, G., Novelli, A., Mei, D., Guerrini, R., Capuano, A., Pantaleoni, C., & Tartaglia, M. (2020). Infantile-Onset Syndromic Cerebellar Ataxia and CACNA1G Mutations. *Pediatric neurology*, ***104***, 40–45.

Berecki, G., Helbig, K. L., Ware, T. L., Grinton, B., Skraban, C. M., Marsh, E. D., Berkovic, S. F., & Petrou, S. (2020). Novel Missense CACNA1G Mutations Associated with Infantile-Onset Developmental and Epileptic Encephalopathy. International journal of molecular sciences, **21(17)**, 6333.

Casas-Alba, D., López-Sala, L., Pérez-Ordóñez, M., Mari-Vico, R., Bolasell, M., Martínez-Monseny, A. F., Muchart, J., Fernández-Fernández, J. M., Martorell, L., & Serrano, M. (2021). Early-onset severe spinocerebellar ataxia 42 with neurodevelopmental deficits (SCA42ND): Case report, pharmacological trial, and literature review. American journal of medical genetics. Part A, **185(1)**, 256–260.

Chemin, J., Siquier-Pernet, K., Nicouleau, M., Barcia, G., Ahmad, A., Medina-Cano, D., Hanein, S., Altin, N., Hubert, L., Bole-Feysot, C., Fourage, C., Nitschké, P., Thevenon, J., Rio, M., Blanc, P., Vidal, C., Bahi-Buisson, N., Desguerre, I., Munnich, A., Lyonnet, S., … Cantagrel, V. (2018). De novo mutation screening in childhood-onset cerebellar atrophy identifies gain-of-function mutations in the CACNA1G calcium channel gene. *Brain : a journal of neurology*, ***141*(7)**, 1998–2013.

Coutelier, M., Blesneac, I., Monteil, A., Monin, M. L., Ando, K., Mundwiller, E., Brusco, A., Le Ber, I., Anheim, M., Castrioto, A., Duyckaerts, C., Brice, A., Durr, A., Lory, P., & Stevanin, G. (2015). A Recurrent Mutation in CACNA1G Alters Cav3.1 T-Type Calcium-Channel Conduction and Causes Autosomal-Dominant Cerebellar Ataxia. *American journal of human genetics*, ***97*(5)**, 726–737.

Gazulla, J., Izquierdo-Alvarez, S., Ruiz-Fernández, E., Lázaro-Romero, A., & Berciano, J. (2021). Episodic Vestibulocerebellar Ataxia Associated with a *CACNA1G* Missense Variant. *Case reports in neurology*, ***13*(2)**, 347–354.

Hara, N., Nezu, T., Kobatake, K., Morino, H., Kawakami, H., & Maruyama, H. (2019). Treatment of intractable resting tremor of spinocerebellar ataxia 42 with zonisamide. *Journal of the neurological sciences*, ***396***, 119–120.

Hashiguchi, S., Doi, H., Kunii, M., Nakamura, Y., Shimuta, M., Suzuki, E., Koyano, S., Okubo, M., Kishida, H., Shiina, M., Ogata, K., Hirashima, F., Inoue, Y., Kubota, S., Hayashi, N., Nakamura, H., Takahashi, K., Katsumoto, A., Tada, M., Tanaka, K., … Tanaka, F. (2019). Ataxic phenotype with altered CaV3.1 channel property in a mouse model for spinocerebellar ataxia 42. Neurobiology of disease, **130**, 104516.

Kimura, M., Yabe, I., Hama, Y., Eguchi, K., Ura, S., Tsuzaka, K., Tsuji, S., & Sasaki, H. (2017). SCA42 mutation analysis in a case series of Japanese patients with spinocerebellar ataxia. *Journal of human genetics*, ***62*(9)**, 857–859.

Kunii, M., Doi, H., Hashiguchi, S., Matsuishi, T., Sakai, Y., Iai, M., Okubo, M., Nakamura, H., Takahashi, K., Katsumoto, A., Tada, M., Takeuchi, H., Ishikawa, T., Miyake, N., Saitsu, H., Matsumoto, N., & Tanaka, F. (2020). De novo CACNA1G variants in developmental delay and early-onset epileptic encephalopathies. Journal of the neurological sciences, **416**, 117047.

Li, X., Zhou, C., Cui, L., Zhu, L., Du, H., Liu, J., Wang, C., & Fang, S. (2018). A case of a novel CACNA1G mutation from a Chinese family with SCA42: A case report and literature review. *Medicine*, ***97*(36)**, e12148.

Morino, H., Matsuda, Y., Muguruma, K., Miyamoto, R., Ohsawa, R., Ohtake, T., Otobe, R., Watanabe, M., Maruyama, H., Hashimoto, K., & Kawakami, H. (2015). A mutation in the low voltage-gated calcium channel CACNA1G alters the physiological properties of the channel, causing spinocerebellar ataxia. Molecular brain, **8**, 89.

Ngo, K., Aker, M., Petty, L. E., Chen, J., Cavalcanti, F., Nelson, A. B., Hassin-Baer, S., Geschwind, M. D., Perlman, S., Italiano, D., Laganà, A., Cavallaro, S., Coppola, G., Below, J. E., & Fogel, B. L. (2018). Expanding the global prevalence of spinocerebellar ataxia type 42. Neurology. Genetics, **4(3)**, e232.

Riquet, A., Cleuziou, P., Floret, V., Quesque, F., Defoort, S., & Smol, T. (2023). Paroxysmal Tonic Upgaze in a Patient With Congenital Ataxia due to a De Novo Missense Variant of CACNA1G. *Pediatric neurology*, ***139***, 22–23.

Tadel F, Baillet S, Mosher JC, Pantazis D, Leahy RM. Brainstorm: a user-friendly application for MEG/EEG analysis. Comput Intell Neurosci 2011
